# A spatially collocated sound thrusts a flash into awareness

**DOI:** 10.3389/fnint.2015.00016

**Published:** 2015-02-27

**Authors:** Máté Aller, Anette Giani, Verena Conrad, Masataka Watanabe, Uta Noppeney

**Affiliations:** ^1^Computational Cognitive Neuroimaging Laboratory, Computational Neuroscience and Cognitive Robotics Centre, University of BirminghamBirmingham, UK; ^2^Max Planck Institute for Biological CyberneticsTübingen, Germany

**Keywords:** multisensory integration, awareness, attention, consciousness, audiovisual, perception, ventriloquism, perceptual illusion

## Abstract

To interact effectively with the environment the brain integrates signals from multiple senses. It is currently unclear to what extent spatial information can be integrated across different senses in the absence of awareness. Combining dynamic continuous flash suppression (CFS) and spatial audiovisual stimulation, the current study investigated whether a sound facilitates a concurrent visual flash to elude flash suppression and enter perceptual awareness depending on audiovisual spatial congruency. Our results demonstrate that a concurrent sound boosts unaware visual signals into perceptual awareness. Critically, this process depended on the spatial congruency of the auditory and visual signals pointing towards low level mechanisms of audiovisual integration. Moreover, the concurrent sound biased the reported location of the flash as a function of flash visibility. The spatial bias of sounds on reported flash location was strongest for flashes that were judged invisible. Our results suggest that multisensory integration is a critical mechanism that enables signals to enter conscious perception.

## Introduction

For effective interactions an organism needs to merge signals from different senses into a coherent and unified percept of the environment. A controversial question is to which extent multisensory integration is automatic or relies on higher cognitive resources such as attention or awareness (for review see Talsma et al., 2010). Even though recent studies have demonstrated that awareness and attention can be dissociated (Koch and Tsuchiya, 2007, 2012; Wyart and Tallon-Baudry, 2008; Watanabe et al., 2011), in many situations attention and awareness are closely intertwined. Hence, for the purpose of this study we do not yet intend to dissociate these aspects, but loosely define “automatic integration” as integration that is relatively immune to attention and awareness. According to the account of automatic integration multisensory co-stimulation increases the bottom-up stimulus saliency (Onat et al., 2007). Thus, signals that co-occur within a spatial and temporal window of integration can automatically amplify stimulus salience. Multisensory integration thereby enables multisensory events to enter perceptual awareness and capture an organism’s attention.

In support of automatic integration a vast body of psychophysics and neurophysiological research has shown that multisensory integration is immune to attentional modulation (Bertelson et al., 2000; Vroomen et al., 2001; Stekelenburg et al., 2004; Bresciani et al., 2006), emerges prior to participants’ awareness (Alsius and Munhall, 2013) and even persists in the anesthetized non-human primate brain (e.g., superior colliculus, primary sensory areas) (Kayser et al., 2005; Stanford et al., 2005). Yet, the account of “automatic” integration has more recently been challenged. For instance, the audiovisual McGurk illusion falters, when attention is diverted to a secondary task (Alsius et al., 2005) or when subjects are unaware of the visual speech gestures (Munhall et al., 2009). Moreover, neuroimaging studies have shown profound attentional modulation of neural multisensory integration indices. Thus, attention modulated the amplification of the BOLD response for congruent audiovisual speech signals in superior colliculi, primary sensory and association cortices (Fairhall and Macaluso, 2009). Likewise, EEG studies showed attentional influences on audiovisual interactions already at ≤100 ms poststimulus (Talsma et al., 2007). With respect to perceptual awareness, the role of primary sensory areas is still debated. While numerous studies have demonstrated that activations in primary sensory areas correlate with participants’ awareness (Tong, 2003), others have suggested that these activations may be mediated by concurrent attentional effects (Watanabe et al., 2011). Collectively, this body of research suggests a multifaceted and not yet completely understood interplay between multisensory integration and higher cognitive processes such as attention or awareness (Talsma et al., 2010).

This intricate relationship partly results from the hierarchical nature of multisensory perception where different types of information (e.g., temporal, spatial, semantic, phonological) are integrated at distinct cortical levels (Bonath et al., 2007; Driver and Noesselt, 2008; Lewis and Noppeney, 2010; Werner and Noppeney, 2010; Lee and Noppeney, 2011, 2014). Conversely, perceptual awareness and attentional capture rely on a cascade of neural processes. Thus, experiments using masking (Chen and Spence, 2011), attentional blink (Soto-Faraco and Spence, 2002; Olivers and Van der Burg, 2008; Adam and Noppeney, 2014), binocular/perceptual rivalry (Hupé et al., 2008; van Ee et al., 2009; Alais et al., 2010; Conrad et al., 2010, 2012, 2013; Lunghi et al., 2010, 2014; Zhou et al., 2010; Guzman-Martinez et al., 2012; Klink et al., 2012; Lunghi and Alais, 2013; Lunghi and Morrone, 2013) or flash suppression (Palmer and Ramsey, 2012; Alsius and Munhall, 2013) are likely to perturb the interplay between perceptual awareness and multisensory integration at different processing stages (for related discussion focusing on visual context, see Fogelson et al., 2014; Peremen and Lamy, 2014; for a recent review see Deroy et al., 2014). In particular, using binocular rivalry numerous studies have demonstrated that a concurrent non-visual signal increases the dominance and decreases the suppression times of the congruent visual percept. Yet, because of the presence of two rivaling percepts, these binocular rivalry experiments make it more difficult to unambiguously determine that the rivalry dynamics was shaped by interactions between the non-visual signals with the suppressed rather than the dominant percept (for further discussion, please see Conrad et al., 2010).

Continuous flash suppression (CFS) is a powerful technique to manipulate participants’ perceptual awareness (Tsuchiya and Koch, 2005). Flashing a mask to one eye can render even a salient stimulus presented to the other eye invisible. Critically, CFS is thought to affect cortical activity already at the primary cortical level via a gain control mechanism (Yuval-Greenberg and Heeger, 2013). CFS thus provides a very useful paradigm to investigate whether a concurrent non-visual signal can counteract the effect of flash suppression at the primary cortical level. Indeed, a previous study has demonstrated that an auditory speech signal makes participants more likely to detect a congruent relative to an incongruent speech video under CFS (Alsius and Munhall, 2013; see also Palmer and Ramsey, 2012). These results suggest that audiovisual synchrony and temporal correlations are important determinants for audiovisual interactions prior to participants’ awareness. Moreover, as natural speech signals evolve continuously over time, temporal expectations may also play an important role in enabling participants to detect visual speech signals.

Yet, as this previous study has presented auditory and visual signals only in a spatially congruent fashion, it could not evaluate the role of spatial congruency, which is another critical cue for multisensory binding. Spatial congruency may enable multisensory interactions via at least two mechanisms. First, spatial congruency may act as a bottom-up cue informing the brain that two signals are likely to come from a common source and should hence be bound into a coherent percept. Second, a spatially collocated sound may reduce the spatial uncertainty about a concurrent flash. Even though spatial congruency affects detection performance only rarely in redundant target paradigm (Forster et al., 2002; Bertini et al., 2008) the second mechanism may be more important in paradigms where the visual signal has been strongly attenuated by various experimental manipulations such as flash suppression or masking. Spatial uncertainty may be reduced via bottom-up mechanisms that enable the formation of more precise audiovisual spatial salience maps. Alternatively, a co-located sound may reduce spatial uncertainty even via top-down expectations that stabilize visual representations potentially even after they have accessed awareness.

Previous studies have demonstrated that a sound increases the detectability of a collocated yet masked visual flash at threshold visibility (Frassinetti et al., 2002; Bolognini et al., 2005). Yet, as these masking studies reduced flash detectability only to threshold performance of 70%, the suppression of awareness for the undetected stimuli was rather shallow. Moreover, it is still unknown whether masking and dynamic CFS reduce visual awareness via similar neural mechanisms (Fogelson et al., 2014; Peremen and Lamy, 2014).

To further investigate the role of spatial congruency in multisensory integration prior to perceptual awareness, the current study combined spatial audiovisual stimulation with dynamic CFS (Tsuchiya and Koch, 2005; Maruya et al., 2008). On each trial, participants were presented with a single flash in the center, their left or right hemifield together with a sound that was spatially congruent or incongruent. Participants located the flash (i.e., flash localization) and judged its visibility (i.e., visual detection task). First, we investigated whether participants were better at detecting the flash when the sound was spatially collocated. We hypothesized that spatial constraints are critical for audiovisual integration processes prior to participants’ awareness. Second, we investigated whether the concurrent sound biased participants’ perceived flash location and whether this bias depended on flash visibility. Importantly, as CFS obliterated visual awareness only in a fraction of trials, we were able to compare the audiovisual spatial bias for physically identical flashes that were visible or invisible.

## Materials and methods

### Participants

After giving informed consent, 24 healthy young adults with normal or corrected-to-normal vision participated in this study (14 females, mean age: 26.7 years, standard deviation: 5.3, range: 18–40; 22 right-handed). One subject was excluded because she did not follow task instructions properly as she located the visual stimuli almost exclusively in the center (98.5%, (group mean ± SD): 35.7% ± 17.5%). The study was approved by the local ethics review board of the University of Tübingen.

### Stimuli and apparatus

Participants sat in a dimly lit room in front of a computer monitor at a viewing distance of 1 m. They viewed one half of the monitor with each eye using a custom-built mirror stereoscope. Visual stimuli were composed of targets and masks that were presented on a gray, uniform background with a mean luminance of 15.5 cd/m^2^. One eye viewed the target stimuli, the other eye the masks.

The target stimuli were three gray discs (Ø 0.29°, mean luminance: 25.4 cd/m^2^), located in the center and 5.72° visual angle to the left and right. On each trial, the color of exactly one of the targets changed to white (mean luminance: 224.2 cd/m^2^) for a duration of 100 ms. This change in brightness will be referred to as “flash”. To suppress the flash’s perceptual visibility, the other eye was shown three dynamic Mondrians (Ø 2°, mean luminance: 35.6 cd/m^2^) (Tsuchiya and Koch, 2005; Maruya et al., 2008). We employed dynamic CFS, as this proved a powerful and reliable method to suppress perceptual awareness of a brief and hence relatively salient flash. To match the target’s location the Mondrians were also located in the center or 5.72° to the left and right of the fixation dot. Each Mondrian consisted of sinusoidal gratings (Ø 0.57°) which changed their color and position randomly at a frequency of 10 Hz. Each grating’s texture was shifted every 16.6 ms to generate apparent motion. Visual stimuli were presented with a fixation spot in the center of the screen and were framed by a gray, isoluminant square aperture of 8.58° × 13.69° in diameter to aid binocular fusion.

Auditory stimuli were pure tones with a carrier frequency of 1 kHz and a duration of 100 ms. They were presented via four external speakers, placed above and below the monitor. Upper and lower speakers were aligned vertically and located 2.3° to the left and 2.3° to the right of the monitor’s center. Speakers’ location was chosen by trading off physical alignment of visual and auditory stimulus locations and sound localization performance. Moreover, it traded off optimization for the two research questions we addressed in this study: (i) the role of audiovisual localization; and (ii) auditory bias on perceived visual location. At a distance of 2.3° mean sound localization accuracy amounted to ~70%.

Psychophysical stimuli were generated and presented on a PC running Windows XP using the Psychtoolbox version 3 (Brainard, 1997; Kleiner et al., 2007) running on Matlab 7 (Mathworks, Nantucket, Massachusetts). Visual stimuli were presented dichoptically using a gamma-corrected 30” LCD monitor with a resolution of 2560 × 1600 pixels at a frame rate of 60 Hz (GeForce 8600GT graphics card). Auditory stimuli were digitized at a sampling rate of 44.8 kHz via an M-Audio Delta 1010LT sound card and presented at a maximal amplitude of 73 dB sound pressure level. Exact audiovisual onset timing was confirmed by recording visual and auditory signals concurrently with a photo-diode and a microphone.

### Experimental design

**Figure 1:**
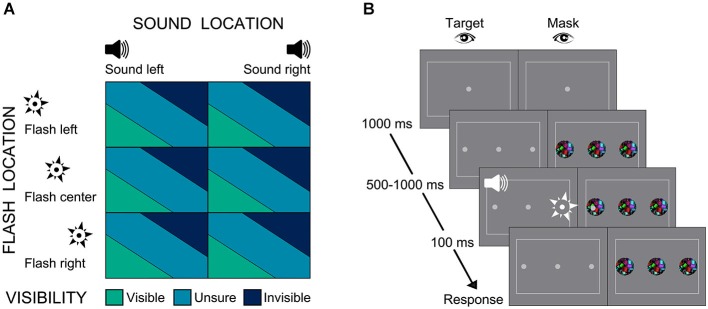
**Experiment paradigm and sample trial. (A)** Experiment design 2 × 3 factorial design with factors: (i) Sound location: left, right; (ii) Flash location: left, center, right. **(B)** Example trial and procedure of dynamic flash suppression.

Participants were presented with an auditory beep emanating from either the left or right. In synchrony with the beep, one eye was presented with a brief flash in the center or participants’ left or right hemifield. The visibility of the flash was suppressed by presenting masks to the other eye using the method of dynamic CFS (Maruya et al., 2008). Hence, the 3 × 2 factorial design manipulated (1) “flash location” (3 levels: left, center, right) and (2) “sound location” (2 levels: left, right) (Figure 1A). On each trial, participants located the flash (left, right or center). Moreover, they performed a graded detection task by judging the visibility of the flash (invisible, unsure, visible).

This experimental design enabled us to address two questions: First, we investigated whether participants were better at detecting the flash, when auditory and visual signals were approximately collocated. Second, as the flash was visible only in a fraction of trials, we were able to quantify the effect of sound on localizing physically identical flashes that were visible or invisible.

### Experimental procedure

As seen in Figure 1B, each trial started with the presentation of the fixation dot for a duration of 1000 ms. Next, participants’ one eye was presented with three gray discs, located in the center, 5.72° visual angle to the left and right. Participants’ awareness of these discs was suppressed by showing dynamic Mondrians at the corresponding locations to the other eye (i.e., dynamic CFS). The Mondrian masks and the discs were presented on the screen until participants had responded to all questions. The assignment of eyes to disks or masks was changed after each trial, to enhance suppression. After a random interval of 500–1000 ms one of the three discs “flashed”, i.e., changed its luminance for a duration of 100 ms. In synchrony with the flash, an auditory beep was played from the left or right. In addition, on 22.2% of the trials, the so-called catch trials, participants were also asked to locate the sound (left vs. right discrimination; in addition to the visibility judgment and flash localization). This allowed us to assess the spatial information that is available for sound localization. Moreover, it ensures that participants did not completely ignore the sound.

Participants responded by pressing one of three buttons on a keyboard. The button assignment was counterbalanced across participants as follows: Participants used three sets of buttons to respond to the three question types (flash localization, sound localization (on catch trials only) and visibility judgment). Each set contained three buttons, one central, one to the left and one to the right. One set of buttons was operated with one hand and the other two sets were operated with the other hand. The association of the hands to the button sets was counterbalanced across participants. Moreover, we also counterbalanced the button response assignment for the flash visibility question. Within subjects we counterbalanced the two possible question orders (i.e., (i) flash localization, (ii) sound localization (only on catch trials), (iii) visibility judgment; alternatively: (i) sound localization (only on catch trials), (ii) flash localization, (iii) visibility judgment).

Prior to the main experiment, participants were familiarized with stimuli and task. First, they completed 2–3 sessions of sound localization. Next, there were two short practice sessions of the main paradigm. During the main experiment participants completed a total of 24 experimental sessions distributed over two successive days, resulting in a total of 1296 trials (i.e., 216 trials per condition).

### Analysis

Our analysis addressed two questions:

#### Effect of spatial congruency on visibility judgment

We investigated whether a synchronous sound boosts “a suppressed visual signal” into participants’ awareness depending on spatial congruency. In other words, we asked whether participants were better at detecting a flash, when the sound was approximately collocated with the flashing disc. Visibility judgment as the dependent variable was quantified as the percentage of non-catch trials judged as visible. As participants’ visibility judgment depended on stimulus eccentricity, we limited this analysis only to those trials with left/right flashes and excluded trials with flashes in the center. Moreover, we pooled over the left and right hemifield as there was no significant difference between left and right hemifield in percentage judged visible. Hence, congruent conditions included flash left/sound left and flash right/sound right combination. Likewise, incongruent conditions included flash left/sound right and flash right/sound left combinations. We performed paired *t*-tests to compare participants’ visibility judgment between congruent and incongruent conditions. However, to be consistent with the statistical analyses used for comparisons concerning the relative auditory weight (detailed in the next paragraph) we also performed a non-parametric bootstrap test based on the one-sample t-statistic for the congruent minus incongruent difference (Efron and Tibshirani, 1993).

#### Effect of sound location on perceived flash location as a function of visibility

We investigated whether the influence of the sound on flash localization depended on the visibility of the flash. Critically, the flash signal intensity was fine-tuned in several pilot studies, so that approximately 50% of the flashes were judged invisible across participants at the group level. Hence, the flash visibility varied across trials and participants because of internal systems noise and participant-specific effects rather than external signal strength. We hypothesized that the influence of the true sound location would be inversely related to flash visibility. In other words, we expected that the influence of the sound on perceived flash location should be maximal for trials where the flash was judged invisible.

To quantify the influence of true sound location on participants’ perceived flash location, we first coded the perceived and true flash and sound locations as −1 for left, 0 for center and 1 for right. Separately for visible, unsure and invisible trials, we then estimated a general linear model where participants’ perceived flash location as the dependent variable was predicted by the true flash and sound location on each trial:

(1) $$V_{p}=\beta_{0}+\left(\beta_{V}*V_{t}\right)+\left(\beta_{A}*A_{t}\right)+\text{ε}$$

with ***V**_p_* = perceived/reported flash location, ***V**_t_* = true flash location, ***A**_t_* = true sound location, *β_0_* = intercept term, *β_V_* = coefficient for true flash location, *β_A_* = coefficient for true sound location, ε = error term. As the audiovisual spatial discrepancies in this experiment were smaller than 10° visual angle, we assumed that auditory and visual signals are combined linearly as assumed under the standard forced fusion model (Alais and Burr, 2004). In other words, the influence of the true sound location (as quantified by the regression coefficient *β_A_*) is assumed not to vary with the spatial discrepancy. Hence, we did not include an interaction term *A_t_* × *V_t_* in the regression model.

We computed the relative auditory weight as an index of the influence of sound on perceived flash location according to:

(2) $$Relative\;Auditory\;Weight=\frac{\beta_{A}}{\beta_{A}+\beta_{V}}$$

We tested whether the relative auditory weight was greater than zero using one-sample *t*-tests. A positive auditory weight indicates that the perceived visual location is shifted towards the true auditory location as expected for a reverse ventriloquist illusion. A negative auditory weight suggests that the perceived visual location is shifted away from the true auditory location (i.e., repulsion effect). An auditory weight that is not significantly different from zero suggests that the location of the sound does not significantly influence the perceived location of the flash. For comparison across visibility levels a one-way repeated measures ANOVA was performed with factor visibility. Planned pairwise comparisons were performed using paired *t*-tests. Moreover, to refrain making any parametric assumptions (n.b. the relative auditory weight conforms to a ratio distribution) we repeated these comparisons using non-parametric bootstrap-based tests.

## Results

### Effect of spatial congruency on visibility judgment

**Figure 2:**
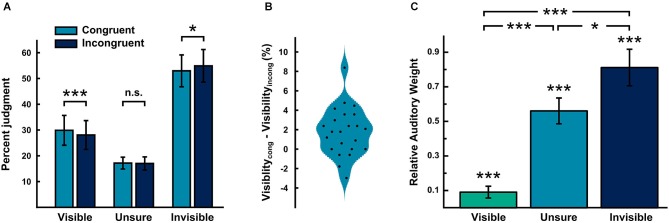
**Behavioral results. (A)** Bar plots showing the percentage of flashes judged visible, unsure and invisible for audiovisual spatially congruent and incongruent conditions (across subjects mean ± SEM). Critically, the % judged visible was significantly higher for audiovisual spatially congruent relative to incongruent conditions. **(B)** Violin plot showing the distribution of the individual differences in percentage of flashes judged visible between the spatially congruent and incongruent conditions. The individual data points are overlaid. **(C)** Bar plots showing the relative auditory weights (across subjects mean ± SEM) obtained from the regression model separately for visible, unsure and invisible trials. As the regression model (specified in the methods) can only be estimated with at least three trials present for a particular visibility level, the number of subjects varies across the different visibility levels (visible: *n* = 21; unsure: *n* = 22; invisible: *n* = 23).

Figure 2A shows the percentage of trials judged visible, unsure and invisible. As expected we observed a significant increase in percentage judged visible, when the sound was presented in the same relative to the opposite hemifield (percentage judged visible: congruent − incongruent (mean ± SEM): 1.8 ± 0.51; Cohen’s d: 0.73; paired-samples *t*-test, *t*_(22)_ = 3.51, *p* = 0.002, bootstrap-based *p* < 0.001) (see Figure 2B for individual differences). Conversely, we observed a significant decrease in percentage judged invisible for spatially congruent relative to incongruent trials (percentage judged invisible: congruent − incongruent (mean ± SEM): 1.94 ± 0.65; Cohen’s d: −0.62; paired-samples *t*-test, *t*_(22)_ = −2.98, *p* < 0.007; bootstrap-based *p* = 0.011). This suggests that a sound influences whether visual signals reach perceptual awareness depending on audiovisual spatial congruency. As we did not include any trials where no flash was presented, we cannot compute the d-prime for the congruent and incongruent conditions or formally dissociate sensitivity and decisional bias. However, as the evaluation of audiovisual spatial congruency obviously entails spatial localization of both flash and sound, it is inconsistent to assume that audiovisual spatial congruency takes effect by influencing the decisional bias in the visibility judgment task. Moreover, had we included trials without a flash to estimate the false alarm rate, we would have still included the same false alarm rate for spatially congruent and incongruent conditions when computing the d-prime. In other words, the % judged visible directly corresponds to the d-primes for congruent and incongruent conditions.

### Effect of sound location on perceived flash location as a function of visibility

We quantified the influence of sound on perceived flash location across visibility levels in terms of the relative auditory weight obtained from the regression approach (see methods). As the regression model specified can only be estimated when at least three trials are present for a particular visibility level, the relative auditory weights are based on a different number of subjects across the different visibility levels (visible: *n* = 21; unsure: *n* = 22; invisible: *n* = 23). Figure 2C shows the relative auditory weights on the perceived location of a visible, unsure and invisible flash. We observed positive relative auditory weights for all three visibility levels. Critically, the relative auditory weights significantly differed across visibility levels (main effect of visibility: *F*_(1.6,29.8)_ = 25.6, MSE = 3.75, *p* < 0.001). More specifically, the relative auditory weight for visible trials was significantly different from that for unsure or invisible trials (paired-*t* test: unsure-visible *t*_(19)_ = 6.54, parametric *p* < 0.001, bootstrap-based *p* < 0.001; invisible-visible *t*_(20)_ = 6.44, parametric *p* < 0.001, bootstrap-based *p* < 0.001; n.b. the degrees of freedom vary as different numbers of subjects could be included, see above). As expected the auditory influence on perceived flash location was greatest when the flash was judged invisible.

## Discussion

Combining spatial audiovisual stimulation and CFS we investigated whether and how signals from different sensory modalities can interact prior to perceptual awareness. CFS is thought to affect visual perception by attenuating neural activity already in primary visual cortices similar to reducing the contrast of the stimulus (Yuval-Greenberg and Heeger, 2013). It is likely that this attenuation of neural activity destabilizes neural representations and prevents them from propagating up the cortical hierarchy thereby obliterating them from perceptual awareness. To measure the effect of a concurrent sound on participants’ visual awareness, we tuned the strength of the visual flash such that it entered participants’ awareness only on a fraction of trials. We then investigated whether the effect of a synchronous sound on participants’ visibility judgment depended on audiovisual spatial congruency. Indeed, our results demonstrate that participants were more likely to detect the flash, when the sound was co-localized than non-collocated with the flash. In support of an “automatic” account of audiovisual integration these results suggest that an aware auditory signal can boost a weak visual signal into participants’ awareness. Critically, the sound was brief and synchronous with the flash across all conditions. Hence, the effects of spatial congruency are unlikely to be explained by a reduction in temporal uncertainty or more precise temporal expectations. Instead they suggest that audiovisual interactions prior to perceptual awareness are governed not only by temporal (as shown by Alsius and Munhall, 2013) but also by spatial constraints. There are at least two mechanisms by which a collocated sound may enhance flash visibility. First, a collocated sound may influence visual perception via bottom-up mechanisms that boost visual salience and enable the formation of spatially more precise salience maps. Second, a collocated sound may reduce visual spatial uncertainty via top-down mechanisms that enable more effective allocation of attentional resources and stabilize visual representations potentially even after they have accessed awareness. In the current paradigm, top-down mechanisms may be less likely because audiovisual signals were presented in synchrony and participants could respond immediately after the flash. Yet, future electrophysiological studies are needed to determine the role of bottom-up from top-down mechanisms in audiovisual interactions during flash suppression.

In sum, our results suggest that audiovisual interactions emerge largely prior to awareness governed by the classical principles of spatial congruency (Stein and Meredith, 1993; Wallace et al., 2004). These interactions in turn enhance stimulus salience and thereby enable a visual signal to elude flash suppression and enter participants’ awareness. A controversial question is whether spatial congruency acts as a fundamental principle of multisensory integration or depends on stimulus characteristics and task-constraints (for excellent review see Spence, 2013). Accumulating evidence from behavioral research suggests that spatial congruency benefits performance predominantly in tasks where spatial information is relevant (e.g., overt or covert spatial orienting—Harrington and Peck, 1998; Arndt and Colonius, 2003; Diederich et al., 2003; Santangelo and Spence, 2008; Spence, 2010), but less so in detection (e.g., redundant target paradigms or identification tasks—Forster et al., 2002; Bertini et al., 2008; Girard et al., 2011). The current study cannot fully exclude that the role of spatial congruency emerges because subjects were engaged in both visibility judgment and spatial localization. Yet, as in previous masking studies (e.g., Frassinetti et al., 2002; Bolognini et al., 2005) an increase in detection performance was also observed in the absence of an additional localization task, spatial task demands do not seem absolutely critical. Instead, we would suggest that concurrent sounds automatically interact with visual signals as a function of spatial discrepancy in low level visual areas thereby amplifying the neural activity and boosting the flash into participants’ awareness. Future studies are needed to further characterize the critical spatial integration window by systematically manipulating the spatial discrepancy of the audiovisual signals under flash suppression. Together with additional EEG and fMRI studies this research line would allow us to further pinpoint the cortical level at which sounds interact with visual processing under flash suppression.

In addition to judging the flash’s visibility participants also located the flash on each trial. As the spatial discrepancy was approximately 8 degrees visual angle, we would expect that a concurrent, yet spatially discrepant sound biases the perceived visual location (Alais and Burr, 2004). The critical question of this study was whether participants’ perceived flash location was influenced by the sound as a function of flash visibility. As expected we observed that the influence of sound location on perceived flash location increased gradually from visible to unsure and invisible trials. This audiovisual spatial bias profile is consistent with the principle of reliability-weighted integration where a stronger weight should be given to the more reliable signal. Indeed, numerous psychophysics and recent neurophysiological studies (Ernst and Banks, 2002; Alais and Burr, 2004; Morgan et al., 2008; Fetsch et al., 2011, 2013) have demonstrated that humans and non-human primates integrate signals weighted by their reliability approximately in accordance with predictions from Maximum Likelihood Estimation. In contrast to these previous studies we did not manipulate the reliability of the external signals. Instead, the flashes were physically identical across all visibility levels. Yet, identical physical signals will elicit neural representations that vary in their reliability across trials because of trial-specific internal systems noise (Faisal et al., 2008). Thus, as the brain does not have access to the true physical reliability of the sensory signals but only to the uncertainty of the internal representations, it is likely that the sensory weights in the integration process depend on both the noise in the environment and the trial-specific noise in the neural system. Thus, our findings suggest that the relative auditory weight in the integration process depends on the reliability of the trial-specific internal representation evoked by the visual signal. For example, if the visual signal is too weak to elude flash suppression and propagate to higher order association areas, “multisensory” representations for instance in parietal areas or response selection processes in frontal areas may be more strongly dominated by auditory inputs (Gottlieb et al., 1998; Macaluso et al., 2003; Macaluso and Driver, 2005; Bisley and Goldberg, 2010). As sensory noise also determines flash visibility, one may also argue that visible flashes bias participants’ perceived sound location via higher order cognitive biasing mechanisms. In other words, if a flash elicits a noisy representation that does not enter participants’ awareness, participants locate the sound purely based on the auditory input. By contrast, if a flash elicits a strong sensory representation that enters awareness, participants’ perceptual decision is biased by the concurrent visual input. Future neurophysiological and neuroimaging studies are required to determine the neural mechanisms underlying this reliability weighting that emerges from internal noise rather than manipulation of external signal strength.

## Conflict of interest statement

The authors declare that the research was conducted in the absence of any commercial or financial relationships that could be construed as a potential conflict of interest.
